# Personalised penetrance estimation for C9orf72-related amyotrophic lateral sclerosis and frontotemporal dementia

**DOI:** 10.1136/bmjno-2024-000792

**Published:** 2024-09-18

**Authors:** Andrew G L Douglas, Alexander G Thompson, Martin R Turner, Kevin Talbot

**Affiliations:** 1 Oxford Centre for Genomic Medicine, Oxford University Hospitals NHS Foundation Trust, Oxford, UK; 2 Nuffield Department of Clinical Neurosciences, University of Oxford, Oxford, UK

**Keywords:** MOTOR NEURON DISEASE, FRONTOTEMPORAL DEMENTIA, NEUROGENETICS

## Abstract

**Background:**

*C9orf72* hexanucleotide repeat expansions are the most common genetic cause of amyotrophic lateral sclerosis (ALS) and frontotemporal dementia (FTD) in European populations. Variable disease penetrance between families presents a challenge for genetic counselling of at-risk relatives and reduces the predictive utility of testing asymptomatic relatives. We have developed a novel model for estimating penetrance in individual families affected by *C9orf72* using available family history information, allowing the calculation of personalised risk estimates.

**Methods:**

Published aggregated age-of-onset data for *C9orf72*-related ALS/FTD were used to generate age-related cumulative relative risks for at-risk relatives within pedigrees. Age-related relative risks are combined with a priori chance of individuals carrying an expansion based on known pedigree information. Penetrance is calculated as a number of affected individuals divided by the sum of cumulative age-related risks of relatives being affected by 80 years.

**Results:**

This method allows family-specific penetrance to be estimated from family history and at-risk relatives’ personalised age-related ALS/FTD risks to be calculated and illustrated graphically. Penetrance reduces as the number and age of at-risk unaffected relatives increases.

**Conclusions:**

Family history remains the best indicator of penetrance in *C9orf72* expansion carriers. Calculating family-specific penetrance can aid genetic counselling by allowing at-risk relatives a more accurate understanding of their individual risk.

## Introduction

 A non-coding hexanucleotide GGGGCC repeat expansion in *C9orf72* causes up to 40% of familial amyotrophic lateral sclerosis (ALS) and up to 25% of familial frontotemporal dementia (FTD) in European ancestry populations.^1^,^3^ Studies identifying *C9orf72* used multigenerational kindreds of affected individuals, meaning expansion carriers appeared at high risk of ALS/FTD and suggesting *C9orf72*-related ALS or FTD (C9-ALS/FTD) had near-complete penetrance by 80 years.^3^,^5^ However, these studies also noted the expansion in up to 7% of apparently sporadic ALS and 6% of sporadic FTD cases, implying reduced penetrance. Expansions have also been identified in control cohorts at unexpectedly high frequencies.^3 6 7^ Based on the known epidemiology of ALS and FTD and frequency of *C9orf72* expansions in these conditions, a population penetrance of 33% has been calculated.^8^ Similar penetrance estimates for ALS risk (not including FTD) of 24.1%–44.3% have also been determined by analyses of *C9orf72* kindreds.^9 10^

Existence of families with autosomal dominant inheritance and apparently sporadic cases shows average disease penetrance figures are not applicable to specific families. Rather, it is becoming clear that *C9orf72* disease penetrance is context-specific and influenced by a range of genetic and environmental factors. Until these are understood and their effects quantified, the best indicator of disease penetrance in a family remains family history data. In this study, we have used family-specific carrier probabilities together with published age-of-onset data for C9-ALS/FTD to develop a novel method for calculating disease penetrance in individual families.

## Methods

Age-of-onset data for C9-ALS/FTD were obtained from Murphy *et al*, a combined cohort study of age-related penetrance of *C9orf72*-related disease that aggregated individual-level age-of-onset data from 40 articles published between 2011 and 2016.^7^ Data were used where diagnosis of ALS or FTD, sex and age of onset were specified. Cumulative incidence was used to determine the proportion of risk to 80 years lived through by at-risk unaffected relatives. CIs were generated by Kaplan-Meier survival analysis calculator (https://www.statskingdom.com/kaplan-meier.html).^11^

In assessed pedigrees, any individuals with ALS or FTD are assumed to carry *C9orf72* expansions and at least one affected relative must be a confirmed carrier. Where the diagnosis is uncertain, separate calculations can be performed if appropriate where the individual is or is not a manifest *C9orf72*-related disease case. Where exact ages are unknown, these must be estimated if used. Since the model relies on the availability of relatives’ information, estimations are preferable wherever possible. The alternative is to exclude such relatives, potentially skewing the result. However, where no tangible information is known about a relative, their contribution to the model should be excluded.

Each at-risk relative’s chance of carrying a *C9orf72* expansion is calculated according to autosomal dominant inheritance. This risk is multiplied by the chance of each unaffected relative having lived to current age (or age at death) as an asymptomatic carrier. An upper limit of 80 years is used since this is the average UK life expectancy and near the upper limit age-of-onset in Murphy *et al.*^7 12^

Penetrance by 80 years (*K*) is calculated as sum of the number (*n*) of affected (*A*) *C9orf72* expansion-positive relatives divided by sum of the number of affected relatives plus summed products of the number (*i*) of unaffected at-risk relatives’ expansion carrier risks (*C*) multiplied by the probability (*p*) of each having lived to their respective ages asymptomatically. This is represented by the following equation:

*K =* (*A_1_+ A_2_ + … + A_n_*) / [(*A_1_+ A_2_ + … + A_n_) +* (*C_1_* **·** *p_1_+ C_2_* **·** *p_2_ + … + C_i_* **·** *p_i_*)]

## Results

From 1171 *C9orf72* expansion-positive cases,^7^ 1132 were used for age-of-onset risk modelling (600 male, 532 female). Male diagnoses comprised 386 (64%) ALS, 40 (6.7%) ALS-FTD, 160 (27%) FTD and 14 (2.3%) FTD-ALS. Female diagnoses comprised 347 (65%) ALS, 36 (6.8%) ALS-FTD, 143 (27%) FTD and 6 (1.1%) FTD-ALS.

**Figure 1:**
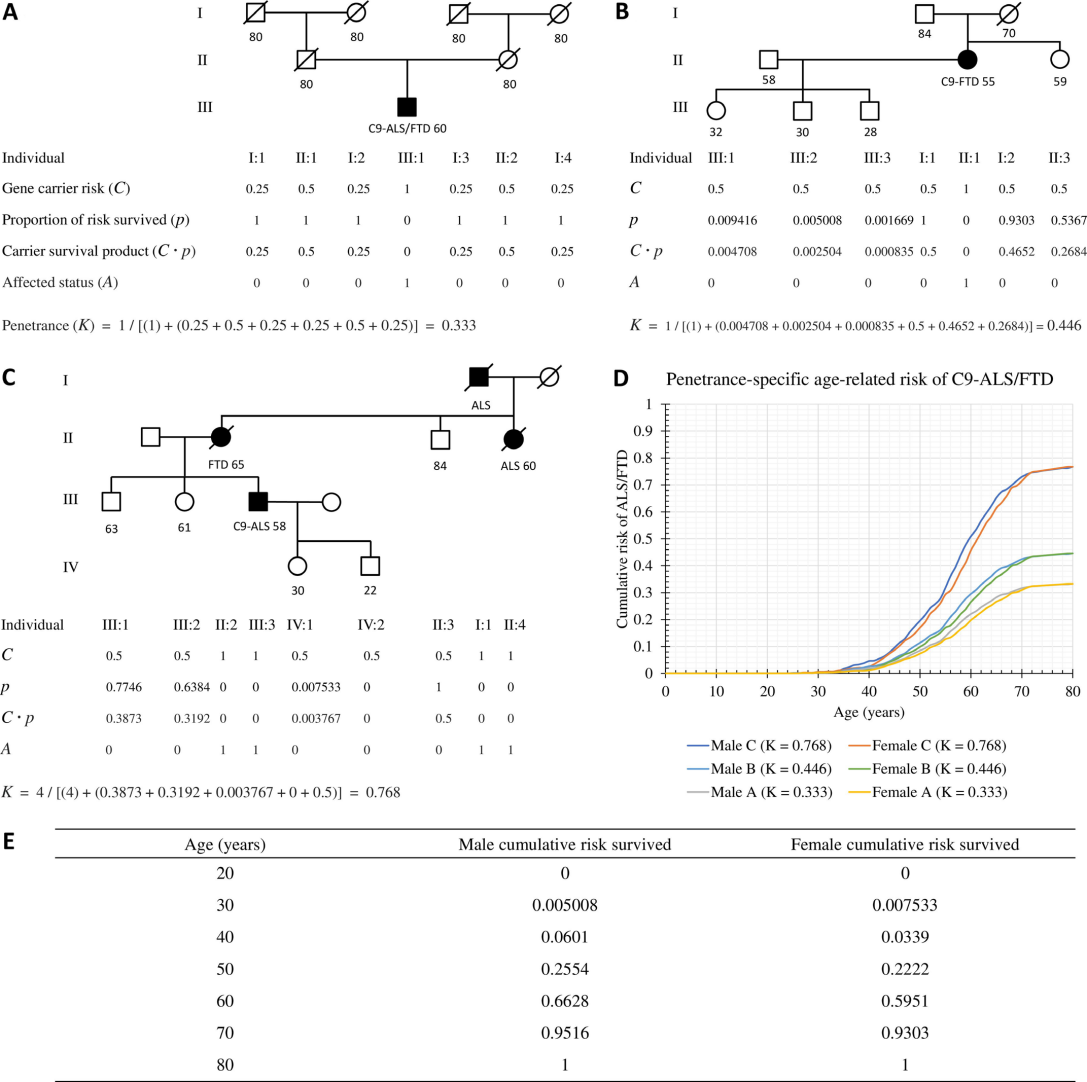
Examples of personalised *C9orf72*-ALS/FTD penetrance calculations based on family history. For each at-risk individual the gene carrier risk (***C***), proportion of risk survived (***p***), and carrier survival product (*C·p*) was determined and from these together with the affected status (***A***) the family disease penetrance (***K***) can be calculated. (**A**). Three-generation idealised pedigree of a sporadic ALS/FTD case where all unaffected relatives lived to 80 years of age. (**B**) Three-generation pedigree of a sporadic FTD case. (**C**). Four-generation pedigree of familial ALS and FTD. (**D**) Age-related cumulative risk of ALS or FTD for males and females up to age 80 is shown for each of the three pedigree scenarios. (**E**) A shortened version of the table for cumulative risk of ALS/FTD lived through by unaffected at-risk males and females depending on age (full table available in online supplemental file 1). ALS, amyotrophic lateral sclerosis; FTD, frontotemporal dementia.

The proportion of cumulative C9-ALS/FTD risk lived through by an at-risk individual is shown in figure 1E, online supplemental figure S1 and online supplemental table S1 and is determined by age-of-onset data. The application of this modelling to specific scenarios is shown in figure 1. Please note these pedigrees are for illustration and are not based on real cases.

Figure 1A shows a three-generation pedigree for a proband with sporadic C9-ALS/FTD aged 60, where parents and grandparents all lived to age 80 unaffected. It is intuitive from autosomal dominant inheritance that disease penetrance in this family is one-third since three individuals must carry the expansion but only one is affected. This penetrance value is confirmed by formal calculation.

Figure 1B illustrates a sporadic case of C9-FTD with one unaffected sibling and three unaffected children. The younger ages of III:1, III:2 and III:3 make their risk contributions small, the main contributors being the proband’s unaffected parents and siblings. This scenario returns a penetrance of 45%. This would reduce to 31% if the proband’s grandparents lived beyond 80 being unaffected and would reduce further still if there were unaffected uncles/aunts.

Figure 1C comprises a four-generation pedigree illustrating a case of familial C9-ALS/FTD with four individuals affected over three generations and five unaffected at-risk individuals of varying ages. While only one affected individual is confirmed to have a *C9orf72* expansion, the other affected relatives are assumed to be carriers. Such a family history would be expected to have high penetrance and the calculation estimates 77%. This would increase to 89% if III:1 and III:2 were to test negative since their risk contributions would then be excluded and would increase to nearly 100% if II:3 were to also test negative. Figure 1D shows the estimated age-related risks for males and females in each of these three pedigrees, the upper limit of risk by age 80 years being the calculated penetrance value (***K***) in each case.

## Discussion

In this report, we describe a new method for estimating ALS or FTD penetrance in individual families carrying *C9orf72* repeat expansions based on family history. By modelling cumulative risk from age-of-onset data combined with family-specific penetrance estimates, lifetime risk for unaffected relatives can be visualised graphically as an aid to understanding personalised risk. Application of this method in clinical settings will facilitate more accurate genetic counselling for C9-ALS/FTD families and allow unaffected relatives to make better-informed decisions in relation to predictive genetic testing, family planning and potential use of presymptomatic therapies. This method is broadly applicable to other genes causing ALS, FTD and similar age-related genetic conditions, provided adequate age-of-onset data are available. While we have not illustrated our model using real family history data, penetrance estimates can be generated for any *C9orf72* family. Since the calculations are essentially numerical translations of pedigree data, there is no practicable confirmatory test to assess family-level method accuracy. However, penetrance estimates generated by this method could in time be used to compare family cohorts and calculate overall penetrance for C9-ALS/FTD.

It should be noted that the amalgamated age-of-onset data from the Murphy *et al* cohort are prone to ascertainment bias. There may also be ethnic and geographic biases, not only on account of *C9orf72* expansions being more common in European populations but also with respect to variability in previous generations’ access to medical diagnoses across populations. Family history is limited by the quality and detail of information available, supplemented by clinically obtainable confirmations and is intrinsically dynamic. Thus, our method only estimates time point-specific penetrance for a given family. Figure 1 illustrates that additional information substantially alters penetrance calculations. Therefore, penetrance values generated by this model should be seen as rough estimates. Furthermore, variability in family data available is case-specific and unquantifiable and we have, therefore, not sought to generate CIs, as this would require subjectivity and might be misleading.

While ALS and FTD are the major phenotypes linked to *C9orf72* repeat expansions, several additional phenotypes are documented, including Alzheimer's disease, Parkinson’s disease, Huntington’s disease-like movement disorder and various psychiatric presentations.^13 14^ Inclusion of such phenotypes adds complexity to pedigree interpretations on account of the high population prevalence of some of these conditions. To minimise the risk of underestimating penetrance, a conservative approach is to assume any such phenotype not otherwise specified in an at-risk relative is related to *C9orf72*.

Genetic and non-genetic factors are believed to play a role in an individual’s ALS and FTD risk. With increasing genomic testing, the effects of genetic modifiers of disease risk should gradually become more tractable. However, other than age, sex and presence of pathogenic variants in known ALS/FTD genes, the relative contributions of most modifying factors have not yet been consistently determined or quantified. Additionally, data about such factors as applied to a consultand’s at-risk relatives are unlikely to be available in the clinical settings. We, therefore, contend our method benefits from practical clinical utility. It is, however, hoped this model can be further refined as the relative contributions of other modifiers are determined. Cancer genetics has benefited from combined risk prediction tools like CanRisk, integrating family data, rare pathogenic variants, polygenic risk, personal and lifestyle factors to generate individualised cancer risk profiles.^15^ As disease-modifying treatments and preventative therapies become available for neurodegeneration, ability to discriminate between high-risk and low-risk individuals will become increasingly relevant.

## Supplementary material

10.1136/bmjno-2024-000792 online supplemental file 1

## Data Availability

All data relevant to the study are included in the article or uploaded as online supplemental information.
