# Supplementary material for: Personalised penetrance estimation for C9orf72-related amyotrophic lateral sclerosis and frontotemporal dementia
Source: BMJ Neurol Open. 2024 Sep 18;6(2):e000792. doi: 10.1136/bmjno-2024-000792 (PMC11418571; doi:10.1136/bmjno-2024-000792)
Supplement: online supplemental file 1 [file bmjno-6-2-s001.pdf]

Supplemental Material:

Personalised penetrance estimation for *C9orf72*-related amyotrophic lateral sclerosis and frontotemporal dementia

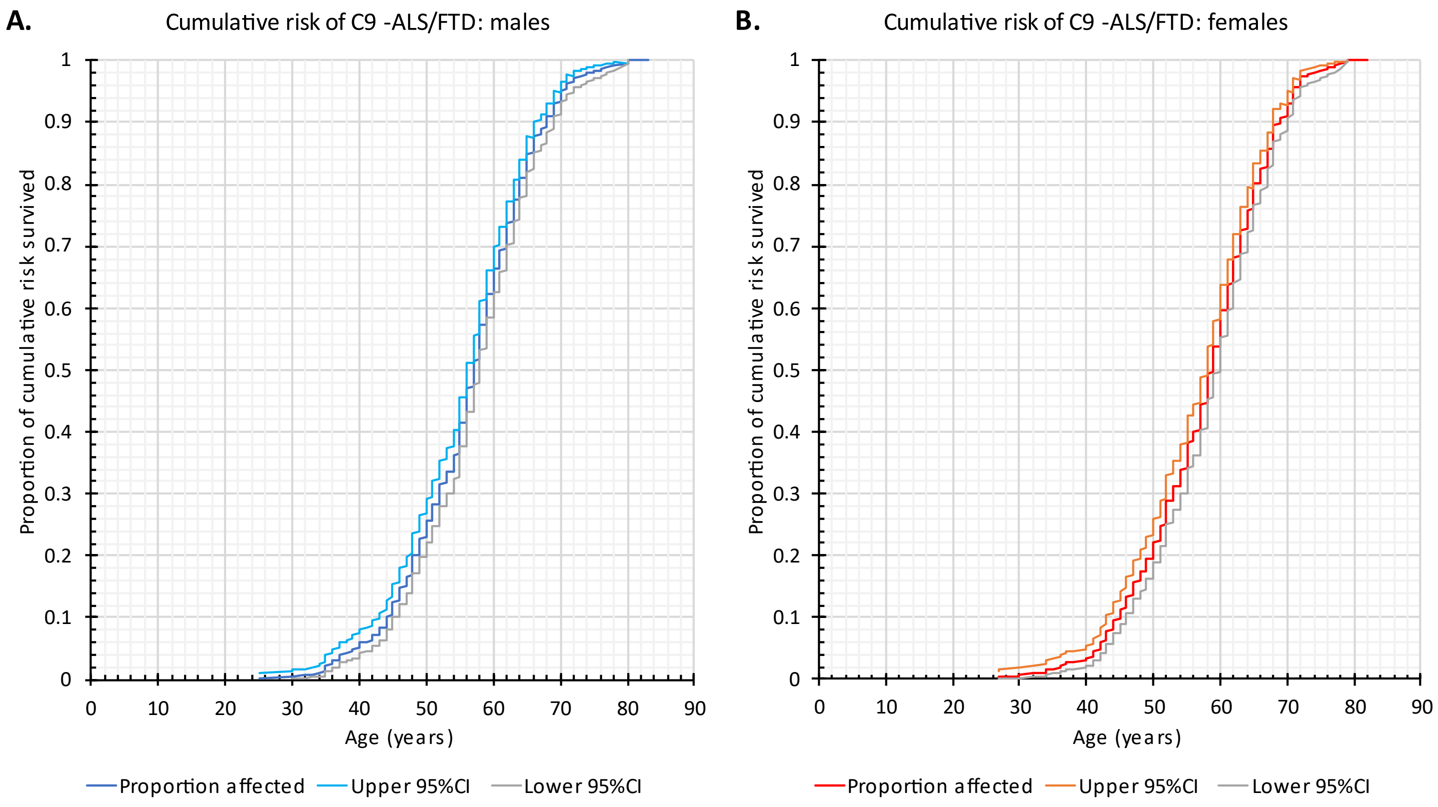

Figure S1. Proportion of cumulative *C9orf72*-ALS/FTD risk lived through by unaffected relatives according to age. **A**. Males. **B**. Females.

Table S1. Calculated C9-ALS/FTD age-related risk values to 80 years for males and females.

Age (years)	Males: risk survived	Males: 95%CI	Females: risk survived	Females: 95%CI
0	0	0 - 0	0	0 - 0
1	0	0 - 0	0	0 - 0
2	0	0 - 0	0	0 - 0
3	0	0 - 0	0	0 - 0
4	0	0 - 0	0	0 - 0
5	0	0 - 0	0	0 - 0
6	0	0 - 0	0	0 - 0
7	0	0 - 0	0	0 - 0
8	0	0 - 0	0	0 - 0
9	0	0 - 0	0	0 - 0
10	0	0 - 0	0	0 - 0
11	0	0 - 0	0	0 - 0
12	0	0 - 0	0	0 - 0
13	0	0 - 0	0	0 - 0
14	0	0 - 0	0	0 - 0
15	0	0 - 0	0	0 - 0
16	0	0 - 0	0	0 - 0
17	0	0 - 0	0	0 - 0
18	0	0 - 0	0	0 - 0
19	0	0 - 0	0	0 - 0
20	0	0 - 0	0	0 - 0
21	0	0 - 0	0	0 - 0
22	0	0 - 0	0	0 - 0
23	0	0 - 0	0	0 - 0
24	0	0 - 0	0	0 - 0
25	0.001669	0.0118 - 0.0002	0	0 - 0
26	0.001669	0.0118 - 0.0002	0	0 - 0
27	0.001669	0.0118 - 0.0002	0.003766	0.015 - 0.0009
28	0.001669	0.0118 - 0.0002	0.003766	0.015 - 0.0009
29	0.001669	0.0118 - 0.0002	0.003766	0.015 - 0.0009
30	0.005008	0.0154 - 0.0016	0.007533	0.0199 - 0.0028
31	0.005008	0.0154 - 0.0016	0.007533	0.0199 - 0.0028
32	0.006678	0.0177 - 0.0025	0.009416	0.0225 - 0.0039
33	0.008347	0.0199 - 0.0035	0.009416	0.0225 - 0.0039
34	0.01169	0.0244 - 0.0056	0.01507	0.0299 - 0.0076
35	0.02337	0.0391 - 0.0139	0.01695	0.0323 - 0.0089
36	0.03005	0.0473 - 0.019	0.0226	0.0395 - 0.0129
37	0.04007	0.0592 - 0.027	0.02637	0.0441 - 0.0157
38	0.04341	0.0631 - 0.0298	0.02825	0.0464 - 0.0171
39	0.05008	0.0709 - 0.0353	0.02825	0.0464 - 0.0171
40	0.0601	0.0823 - 0.0437	0.0339	0.0533 - 0.0215

41	0.06177	0.0842 - 0.0451	0.0452	0.0667 - 0.0305
42	0.07179	0.0956 - 0.0537	0.06026	0.0841 - 0.043
43	0.08347	0.1086 - 0.0639	0.07721	0.1034 - 0.0574
44	0.1018	0.1289 - 0.0802	0.09605	0.1244 - 0.0739
45	0.1252	0.1545 - 0.1012	0.113	0.1431 - 0.0889
46	0.1486	0.1797 - 0.1225	0.1337	0.1657 - 0.1075
47	0.1669	0.1993 - 0.1394	0.1582	0.1921 - 0.1298
48	0.2003	0.2347 - 0.1704	0.1733	0.2082 - 0.1436
49	0.2287	0.2645 - 0.1971	0.194	0.2302 - 0.1628
50	0.2554	0.2923 - 0.2224	0.2222	0.26 - 0.1892
51	0.2821	0.32 - 0.2479	0.2486	0.2876 - 0.214
52	0.3155	0.3544 - 0.28	0.2881	0.3287 - 0.2516
53	0.3356	0.3749 - 0.2993	0.3107	0.352 - 0.2733
54	0.3623	0.4022 - 0.3252	0.339	0.381 - 0.3005
55	0.414	0.4546 - 0.3758	0.3823	0.4251 - 0.3425
56	0.4708	0.5116 - 0.4317	0.4011	0.4442 - 0.3609
57	0.5159	0.5564 - 0.4765	0.4444	0.4878 - 0.4034
58	0.5726	0.6125 - 0.5334	0.4953	0.5386 - 0.4537
59	0.6227	0.6615 - 0.584	0.5367	0.5796 - 0.495
60	0.6628	0.7004 - 0.6248	0.5951	0.637 - 0.5537
61	0.6945	0.731 - 0.6573	0.6384	0.6792 - 0.5976
62	0.7379	0.7724 - 0.7022	0.6817	0.721 - 0.6419
63	0.7746	0.8072 - 0.7404	0.7269	0.7641 - 0.6885
64	0.8097	0.84 - 0.7773	0.7589	0.7944 - 0.7218
65	0.8497	0.877 - 0.8199	0.8004	0.8332 - 0.7654
66	0.8781	0.90281 - 0.8505	0.8249	0.8558 - 0.7913
67	0.8898	0.9133 - 0.8632	0.8569	0.8851 - 0.8256
68	0.9098	0.93107 - 0.8852	0.8964	0.9205 - 0.8687
69	0.9316	0.94991 - 0.90946	0.9077	0.93042 - 0.8812
70	0.9516	0.96678 - 0.93232	0.9303	0.94989 - 0.90657
71	0.9633	0.97627 - 0.94599	0.9567	0.97172 - 0.93696
72	0.9716	0.9828 - 0.95597	0.9736	0.98487 - 0.95731
73	0.975	0.98534 - 0.96004	0.9774	0.98763 - 0.96199
74	0.98	0.98903 - 0.96624	0.9812	0.990305 - 0.96674
75	0.9833	0.991399 - 0.97046	0.9849	0.992864 - 0.97159
76	0.9866	0.993667 - 0.97477	0.9887	0.995269 - 0.97658
77	0.99	0.9958 - 0.97919	0.9925	0.997443 - 0.98175
78	0.9933	0.997729 - 0.98378	0.9962	0.9992162 - 0.98718
79	0.9933	0.997729 - 0.98378	1	1 - 1
80	1	1 - 1	1	1 - 1
